# Cost-effectiveness of a smartphone Application for Tinnitus Treatment (the CATT trial): a study protocol of a randomised controlled trial

**DOI:** 10.1186/s13063-022-06378-7

**Published:** 2022-05-23

**Authors:** Sara Demoen, Laure Jacquemin, Annick Timmermans, Vincent Van Rompaey, Olivier Vanderveken, Hanne Vermeersch, Iris Joossen, Julie Van Eetvelde, Winfried Schlee, Wim Marneffe, Janis Luyten, Annick Gilles, Sarah Michiels

**Affiliations:** 1 Rehabilitation Research Center, REVAL, faculty of Rehabilitation Sciences, Hasselt University, 3500 Hasselt, Belgium; 2 Department of Otorhinolaryngology and Head and Neck Surgery, Antwerp University Hospital, 2650 Edegem, Belgium; 3 Department of Translational Neurosciences, Faculty of Medicine and Health Sciences, University Antwerp, 2000 Antwerp, Belgium; 4 Department of Psychiatry and Psychotherapy, University of Regensburg, 93053 Regensburg, Germany; 5 Faculty of Business Economics, Hasselt University, 3500 Hasselt, Belgium; 6 Department of Human and Social Welfare, University College Ghent, 9000 Ghent, Belgium

**Keywords:** Somatic tinnitus, Smartphone application, Blended physiotherapy, Telerehabilitation, Randomised controlled trial

## Abstract

**Background:**

Tinnitus is a highly prevalent symptom, affecting 10–15% of the adult population. Tinnitus influenced by alterations in somatosensory afference from the neck or jaw is referred to as somatic tinnitus (ST). ST is known to respond positively to physiotherapy treatment; however, it is challenging to motivate patients to systematically perform home exercises correctly, and the necessary tinnitus counselling is often lacking.

The aim of this study is twofold, namely to investigate both the effectiveness and cost-effectiveness of a blended physiotherapy program for ST, including a smartphone application designed to increase exercise therapy compliance and provide tinnitus counselling.

**Methods:**

This study is designed as a single-blind two-arm 1:1 randomised controlled trial (RCT). Adult patients diagnosed with ST, without psychiatric comorbidities and with experience in using a smartphone, will be recruited at the Ear Nose Throat (ENT) department of the Antwerp University Hospital (UZA). Patients will be randomised into two groups. The experimental group will receive the blended physiotherapy program comprising six in-clinic physiotherapy sessions over a period of 12 weeks (1x/2 weeks) and an exercise and counselling program provided by the smartphone application. The control group will receive the standard care program comprising twelve weekly in-clinic physiotherapy sessions. Each physiotherapy session has a duration of 30 min. The primary outcome measure is the change in Tinnitus Functional Index (TFI) score. Additionally, a cost-effectiveness analysis will be performed from a societal perspective considering both direct and indirect costs. There will be follow-up assessments at one and 3 months after the final treatment session.

**Discussion:**

Our study is the first to combine both tinnitus counselling and neck/jaw treatment provided by a digital application in a blended physiotherapy program. This, in order to empower ST patients to improve and better manage their own health and, possibly, reduce economic costs by alleviating the tinnitus burden that ST patients experience. The strengths of the planned RCT are the high-quality methodological design, the large sample size and the expertise of the involved multidisciplinary research team.

**Trial registration:**

Clinicaltrials.gov NCT05245318. Registered on 26 January 2022.

**Supplementary Information:**

The online version contains supplementary material available at 10.1186/s13063-022-06378-7.

## Background

Tinnitus or ringing in the ears is a very common symptom with a variety of possible underlying diseases and/or malfunctions which may be otological, neurological, metabolic, psychogenic and/or somatic in origin [1–3]. It affects 10 to 15% of the adult population and has a widely varying clinical presentation due to the heterogeneity of underlying causes, associated symptoms and comorbidities [4–6]. In 5–10% of tinnitus patients, the symptom is associated with mood changes, anxiety, depression, sleep disorders, concentration problems and other psychological/emotional issues that lead to severe disruptions of daily functioning and health-related quality of life (HRQOL) [7] (Demoen S, Cardon E, Jacquemin L, Timmermans A, Van Rompaey V, Vanderveken O, Vermeersch H, Joossen I, Van Eetvelde J, Schlee W, Marneffe W, Luyten J, Van De Heyning P, Michiels S, Gilles A. Health-related quality of life in subjective, chronic tinnitus patients: a systematic review. Under review). Alleviation of tinnitus can improve the quality of life of many patients and, as a consequence, can reduce the economic burden of tinnitus on society. The general societal cost of tinnitus is extremely high [8, 9]. Three types of costs can be distinguished. Firstly, the direct health care costs that are related to consultations or treatment sessions by healthcare professionals and medication on prescription. Secondly, patient and family costs are additional out-of-pocket costs for the patient or his/her family, such as travel expenses or costs of over-the-counter medication. Lastly, indirect costs are considered the societal costs due to, for example, productivity loss and absence at work [8, 9]. Based upon studies from both USA and Europe, the mean annual costs per patient are estimated between 1544 euro and 3429 euro for healthcare costs, between 69 euro and 115 euro for patient and family costs and between 2565 euro and 3702 euro for indirect costs [8]. To frame, approximately 65 million adults in the European Union suffer from tinnitus, out of which 26 million patients experience bothersome tinnitus and 4 million patients severe tinnitus [10]. Especially patients with bothersome and severe tinnitus seek help, the total costs for this group of 30 million patients is estimated to be between 125 to 217 billion euros on a European level [10].

Among the many potential influencing factors of tinnitus, one group exists where the tinnitus is influenced by alterations in somatosensory afference from the cervical spine or temporomandibular area, caused by, for instance, increased muscle tension or movement restrictions. This type of tinnitus is called somatic or somatosensory tinnitus (ST) and is estimated to be present in approximately 25% of tinnitus patients [11–13]. Previous studies showed that a multimodal physiotherapy treatment directed to cervical spine and temporomandibular joint (TMJ) dysfunctions can provide tinnitus relief in ST patients [14–22]. The multimodal physiotherapy treatment comprises a combination of (home) exercises, manual mobilizations and techniques to decrease muscle tension tailored to the patient’s specific cervical spine and/or TMJ dysfunction [14–17].

While the effectiveness of this multimodal physiotherapy program has been proven, there are still opportunities for improvement. Firstly, the efficacy of the home exercises could be increased, as two thirds of patients are not compliant in performing daily home exercises, and others experience difficulties to perform the exercises correctly without the guidance of a physiotherapist [23]. Secondly, the associated symptoms and comorbidities of tinnitus often demand a more multidisciplinary approach [4–7]. Apart from the physiotherapy program, patients would also benefit from additional psychological counselling and advice to account for the mental burden that comes with tinnitus [12]. Due to a lack of knowledge about tinnitus, physiotherapists often do not provide the additional tinnitus counselling, or patients receive inaccurate advice that may counteract the positive effects of the multimodal treatment. This results in suboptimal care for ST patients and often recurrent complaints that require regular treatment series in the long term. As such, ST patients would benefit from additional support to increase exercise compliance on the one hand and from the addition of psychological counselling on the other hand [4–7, 12, 23].

One way to provide these extra elements is through a smartphone application. In the last decade, computer and smartphone applications increasingly found their way into medicine and physiotherapy treatment. Recently, the COVID-19 pandemic has shown that low-contact treatment provided from a distance through applications and videoconferencing can be used as a substitution of or addition to face-to-face clinical care for several conditions [24–26]. Telerehabilitation and internet-based psychological counselling have already proven to be as effective as standard practice in relieving pain and disability in a variety of musculoskeletal conditions [27, 28] and in reducing distress in case of tinnitus [29]. The use of a smartphone application might provide additional support to motivate the patient to perform daily exercises in order to increase exercise compliance. Furthermore, it can be used to provide psychological tinnitus counselling.

Therefore, the aim of this single-blind non-inferiority two-arm parallel group 1:1 randomised controlled trial is to investigate the effectiveness and cost-effectiveness of a blended physiotherapy program for ST, including a smartphone application designed to increase therapy compliance and provide tinnitus counselling. It is hypothesised that the blended physiotherapy program will at least be as effective as the standard physiotherapy care and that this new intervention will reduce both direct and indirect costs related to ST.

## Methodology

This protocol is reported according to the Standard Protocol Items Recommendations for Interventional Trials (SPIRIT) guidelines 2013: Explanation and Elaboration: guidance for protocols of clinical trials [30].

### Patients

Adult patients (+ 18 years old) can be included in the study in case they are diagnosed with ST, according to the diagnostic criteria for ST, listed in Table 1 [31]. The diagnosis will be made by an experienced multidisciplinary team of Ear Nose Throat (ENT) specialists, audiologists, physiotherapists and psychiatrists at the University Hospital of Antwerp (UZA) (Belgium), in order to identify and take into account all potential influencing factors of a patient’s tinnitus. Patients will only be diagnosed with ST in case the somatosensory influence on the tinnitus is one of the major influencing factors. Tinnitus patients who present themselves with one or more of the ‘Accompanying symptoms’ described in Table 1 are considered suffering from ST in case their tinnitus appeared or aggravates simultaneously with the neck or jaw symptoms. Additionally, in case the occurrence of the tinnitus is closely related to a neck or head trauma, patients are considered to suffer from ST [32].

**Table 1:** Diagnostic criteria for somatic tinnitus (ST) listed by a group of ST experts [31]

**Tinnitus modulation**	
• By voluntary movements of head, neck, jaw or eyes	
• By somatic manoeuvres	
• By pressure on myofascial trigger points	
**Tinnitus characteristics**	
• Simultaneous appearance of tinnitus and neck or jaw pain	
• Simultaneous aggravation of tinnitus and neck or jaw pain	
• Preceded neck or head trauma	
• Increasement tinnitus during certain postures	
• Varying tinnitus pitch, loudness and/or location	
• For unilateral tinnitus, the audiogram does not account for unilateral tinnitus	
**Accompanying symptoms**	
• Frequent pain in the cervical spine, head or shoulder girdle	
• Presence of pressure tender myofascial trigger points	
• Increased muscle tension in the suboccipital muscles	
• Increased muscle tension in the extensor muscles of the cervical spine	
• Temporomandibular disorders	
• Teeth clenching or bruxism	
• Dental disease	

Patients should be fluent in Dutch, in order to complete all questionnaires and perform all tests (including speech-in-noise tests). Additionally, all included patients should own a smartphone and should be able to use the most common applications without support. Patients with other types of tinnitus, active middle ear pathology or diagnosed psychiatric disorders will be excluded from the study.

### Study design and randomisation procedure

**Fig. 1:**
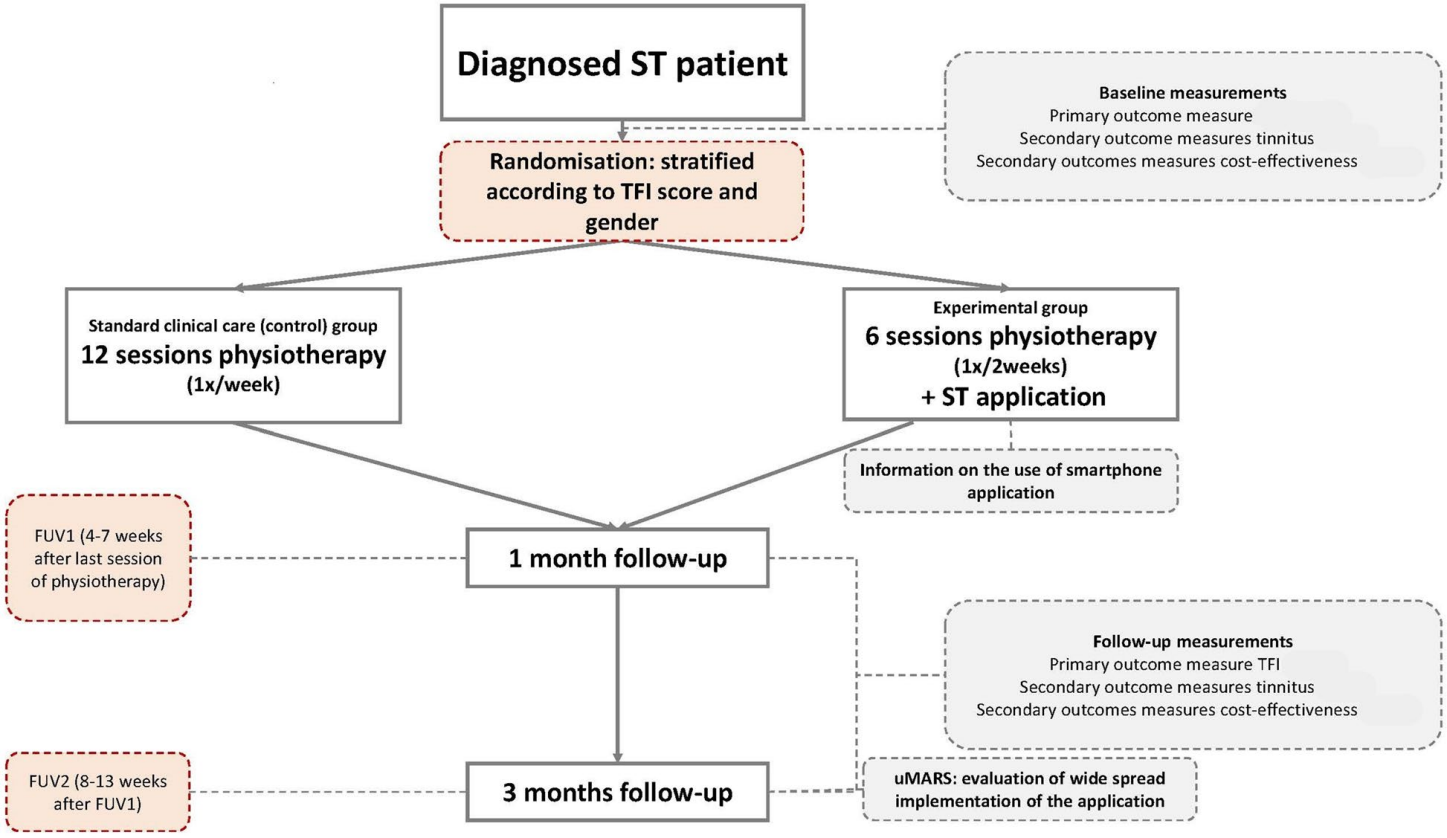
Study flowchart. TFI, Tinnitus Functional Index; ST, somatic tinnitus; FUV, follow-up visit; uMARS, user version of Mobile Application Rating Scale

The study is designed as a single-blind two-arm 1:1 randomised controlled trial. After baseline measurements, patients will be randomised into either the experimental group or the control group. A stratified randomisation according to the grade of tinnitus severity obtained by use of the Tinnitus Functional Index (TFI) and gender will be used. The TFI categorises patients from grade 1 (no tinnitus distress) to grade 5 (very severe tinnitus distress) [33–35]. Only grades 2–5 are considered for this study: light distress (7 to 28), moderate distress (29 to 47), severe tinnitus distress (48 to 65) and very severe tinnitus distress (66 to 100) [35]. The stratified randomisation assures an even distribution of patients of each tinnitus grade and of men and women in both treatment arms. A minimisation procedure will be used to perform the stratified randomisation using the web-based online randomisation tool Qminim [36]. Patients will be informed that the study investigates the effectiveness of two different physiotherapy approaches for ST but will not receive in-detail information about the content of the treatment before randomisation. Hence, patients are blinded towards the group they are randomised to. The therapist cannot be blinded. To limit the risk of bias related to lack of blinding of the therapist, all evaluation measurements will be performed by a blinded assessor and data analysts will be blinded as well. An overview of the study design and randomisation procedure is presented in Fig. 1.

### Outcome measures

**Fig. 2:**
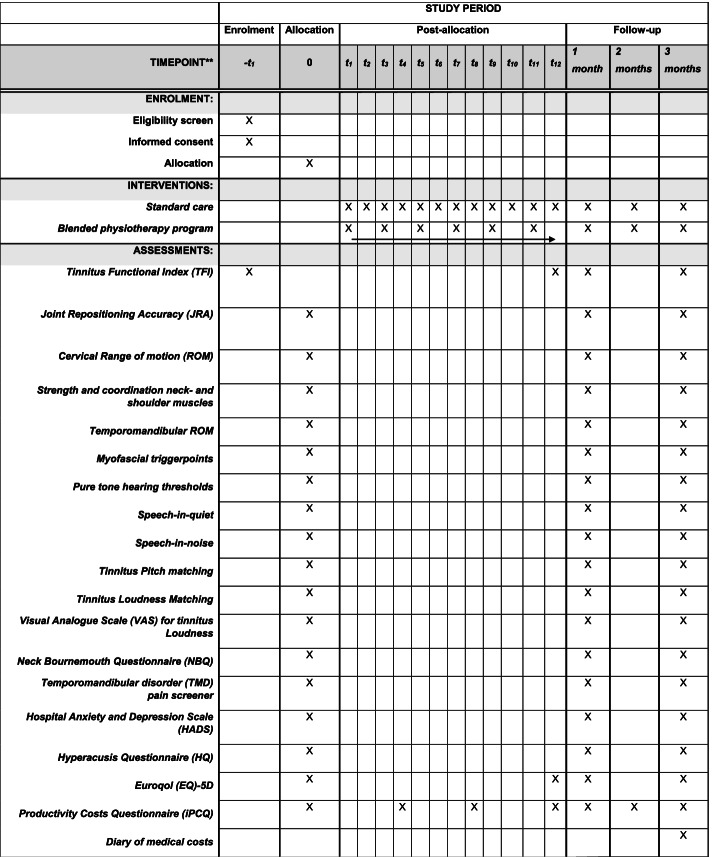
Schedule of enrolment, interventions and assessments in accordance with the SPIRIT 2013 guidelines

The following outcome measures will be collected at baseline and during the two follow-up visits. The first follow-up visit is planned 1 month (range: 4 to 7 weeks) after the last treatment session. The second follow-up visit will take place 3 months (range: 8 to 13 weeks) after the last treatment session. In between the first and second follow-up moment should be a minimum period of 4 weeks. For certain outcome measures additional measuring moments are planned, these are specified individually below. See Fig. 2 for an in-detail schedule of enrolment, interventions and assessments in accordance with the SPIRIT 2013 guidelines [30].

#### Primary outcome measure

The primary outcome is the change in Tinnitus Functional Index (TFI) score from baseline to 1 month after the treatment. This time point was chosen since previous research indicated that the largest treatment effect on TFI is to be expected 1 month after the last treatment session [37–40]. For the cost-effectiveness analysis, an additional time point, immediately after the last treatment session, is added to be able to link the amount of decrease on TFI to the EuroQoL (EQ)-5D-5L that assesses HRQOL. The TFI is a self-report questionnaire comprising 25 questions, each scored on an eleven-point Likert scale, objectifying the impact and severity of tinnitus. Eight subscores are differentiated, namely intrusiveness, sense of control, cognitive complaints, sleep disturbance, auditory difficulties, relaxation, quality of life (QOL) and emotional distress. The total score and subscores will be noted on a scale of 0 to 100 with higher scores indicating higher levels of tinnitus-related distress [33, 34]. A change of 13 points out of 100 is considered a clinically relevant change [33]. TFI has a high sensitivity to change, a good test-retest reliability (*r*: 0.78), discriminant validity with the Beck Depression Inventory-Primary Care (*r*: 0.56) and convergent validity with both the Tinnitus Handicap Inventory (THI) (*r*: 0.86) and the visual analogue scale (VAS) for tinnitus loudness (*r*: 0.75) [33, 41, 42].

#### Secondary outcome measures

##### Clinical cervical and temporomandibular outcome measures

The following tests will be used to assess the dysfunction of the cervical spine and temporomandibular area. Firstly, cervical spine mobility and joint repositioning accuracy (JRA) will be measured using the NeckCare Unit (https://www.neckcare.com/). This device, using accelerometer and gyroscope data, is specifically designed for investigating the cervical range of motion and JRA. Additionally, the strength and coordination of the deep neck flexor and extensor muscles and the shoulder stabilising muscles will be assessed according to the standardised protocol proposed by Segarra et al. [43].

The mobility of the temporomandibular joint will be measured using a ruler. Mouth opening, protrusion and laterotrusion will be objectified in this manner. Finally, active myofascial trigger points will be looked for in the masseter and temporalis muscle and in the sternocleidomastoid, splenius capitis, upper trapezius and levator scapulae muscles [44, 45].

##### Audiological outcome measures

- Pure tone hearing thresholds

Pure tone audiometry, to objectify the presence of hearing loss, will be performed according to current clinical standards (ISO 8253-1, 1989) using a two-channel Interacoustics AC-40 audiometer in a silent room. Air conduction thresholds are measured by the use of headphones at 125 Hz, 250 Hz, 500 Hz, 1 kHz, 2 kHz, 3 kHz, 4 kHz, 6 kHz and 8 kHz. In cases where air conduction thresholds exceed the normality level of 20 dB HL at one or more frequencies between 250 Hz and 4 kHz, bone conduction thresholds will be measured at 250 Hz, 500 Hz, 1 kHz, 2 kHz, 3 kHz and 4 kHz.

- Speech-in-quiet (SPIQ) and speech-in-noise (SPIN) understanding

Speech reception in quiet (SPIQ) will be measured using the Dutch open-set NVA lists developed by the Nederlandse Vereniging voor Audiologie (NVA) (Dutch Society for Audiology) [46]. Each list consists of twelve monosyllabic words (consonant-vowel-consonant), of which one is a training item. The speech recognition score is the percentage of correctly identified phonemes. The lists will be presented through headphones. The speech reception in noise (SPIN) will be assessed by means of the Leuven Intelligibility Sentences Test (LIST) using an adaptive procedure [47]. The frequency spectrum of the noise signal is equal to the long-term average speech spectrum of the sentences. The level of the noise is fixed at 65 dB SPL, while the level of the speech signal is altered depending on the response of the patient. If the participant repeats the keywords of the sentence correctly, the level of the next sentence is decreased by 2 dB SPL. If the participant fails to repeat the keywords correctly, the level is increased by 2 dB SPL. Each list consists of ten sentences, and the speech reception threshold (SRT) is calculated as the mean level of the last five sentences together with the level of the imaginary 11th sentence of the list. This speech in noise test is performed using headphones.

- Psychoacoustic tinnitus measures

**Tinnitus pitch matching**

The pitch is the psychoacoustic equivalent of the physical parameter frequency. The tinnitus pitch is obtained by use of a pitch matching technique which is the quantitative and qualitative description of the spectral characteristics of the tinnitus. For this technique, a two-alternative forced-choice procedure will be used using the contralateral ear as the reference ear. In cases where tinnitus is perceived bilaterally, the choice of the ear is the side where the tinnitus is perceived the loudest. By this technique, an attempt is made to identify the centre pitch of the tinnitus. When multiple tinnitus sounds are perceived, it is suggested to concentrate on the most troublesome tinnitus sound. Each time, a pair of pure tones (or noises in case of noise-like tinnitus), differing by one or more octaves, are presented to the subject, who has to indicate which of the tones resembles the tinnitus the most. This procedure is repeated, and finer adjustments are made to obtain a match of tinnitus pitch as exact as possible.

**Tinnitus loudness matching**

Loudness is the perceptual correlate of the sound intensity. The tone (or noise) defined as the pitch math is presented to the ipsilateral ear (when appropriate), and a loudness match is made by the use of an alternating forced-choice procedure with an accuracy of 1 dB.

##### Questionnaires related to the effectiveness analysis

- VAS for tinnitus loudness

Patients are asked to score the mean and maximum loudness of their tinnitus in the previous week on a 100-mm horizontal line ranging from left: 0 (absence of tinnitus) to right: 100 (as loud as possible, cannot be any louder) [48].

- Neck Bournemouth Questionnaire (NBQ) score

The presence and severity of neck complaints will be evaluated using the NBQ. The NBQ [49] consists of seven questions on the severity of the neck complaints and its interference with the patient’s wellbeing and professional and daily activities. Each item is scored on an eleven-point Likert scale. A higher score out of the maximum of 70 represents higher levels of neck pain and disability [49]. Scores from 13 points upwards are considered to represent a clinically important neck dysfunction [49]. The test-retest reliability of the NBQ is moderate (ICC: 0.65). The construct validity is acceptable with both the Neck Disability Index (*r*: 0.50) and the Copenhagen Neck Functional Index (*r*: 0.44). The effect size was high (Cohen’s *d*: 1.67), which indicates that the NBQ is highly responsive to changes in cervical spine complaints [49–51]. The clinically relevant change of the NBQ is a 12 points decrease [49, 50].

- Temporomandibular disorder (TMD) pain screener score

The presence and severity of temporomandibular disorders (TMD) will be evaluated using the TMD pain screener. The TMD pain screener is a 6-item questionnaire regarding pain complaints from the orofacial region and their dependency on functions, like opening wide or chewing. One item has three answer options, scored zero, one or two. The five remaining items are answered dichotomously with yes or no, respectively scored as one or zero. The minimal score is zero and the maximal score is seven. In case a patient scores at least a three out of seven on the TMD pain screener, the patient is suspected to suffer from a painful TMD (sensitivity 0.99 and specificity 0.95–0.98, respectively) [52]. Internal consistency of the questionnaire is excellent, with a coefficient *α* value of 0.93, reliability is good (ICC: 0.79), and excellent sensitivity and specificity for diagnosing TMD (0.99 and 0.95-0.98, respectively) [51, 53].

- Hospital Anxiety and Depression Scale (HADS)

The Hospital Anxiety and Depression Scale (HADS) is used to detect signs of depression and anxiety symptoms [54]. It is a self-assessment scale and was developed to identify the possibility and probability of the presence of anxiety and depression among patients in non-psychiatric clinics [54]. It exists of two subscales, an anxiety subscale (HADS-A) and a depression subscale (HADS-D), both containing seven intermingled items. Each subscale is scored out of a total of 21; a score greater than eight indicates the presence of anxiety or depression symptoms, respectively [54]. A high internal consistency was stated for a both the depression and anxiety scale, with respectively a mean coefficient *α* value of 0.83 and 0.82 [55]. In addition, both scales also demonstrated a good sensitivity and specificity of approximately 0.80 [55]. The HADS is found to be an instrument of moderate reliability (ICC: 0.56) for screening states of depression and anxiety in the setting of a hospital medical outpatient clinic [51, 54, 55].

- Hyperacusis Questionnaire (HQ)

Hyperacusis, a symptom that often co-occurs with tinnitus, is quantified and characterised using the Dutch version of the Hyperacusis Questionnaire (HQ) [47, 56]. This questionnaire consists of 14 questions that are answered on a 4-point scale, ranging from ‘No’ (0 points), ‘Yes, a little’ (1 point), ‘Yes, quite a lot’ (2 points) to ‘Yes, a lot’ (3 points). Scores on the HQ consequently range from 0 to 42, and the traditional cut-off value for hyperacusis is 28 points [47]. Moderate correlations between the HQ, uncomfortable loudness levels and other health questionnaires and a high internal consistency of the HQ have been demonstrated [57].

##### Questionnaires related to the cost-effectiveness analysis

- EuroQoL (EQ)-5D [58]

The EuroQoL EQ-5D questionnaire is the most commonly used method to collect health-related Quality of Life (HRQOL) data and is recommended by worldwide economic evaluation guidelines such as the NICE recommendations [59, 60]. This questionnaire measures HRQOL using 5 dimensions: mobility, self-care, usual activities, pain/discomfort and anxiety/depression [58]. The response pattern produces a code which represents a health state of the participant. Each health state is associated with a health utility value between 0 and 1 in which 0 represents death and 1 corresponds to perfect health [60]. To calculate utility values, the recently published utility value sets for Belgium will be used [61]. HRQOL is necessary to calculate quality-adjusted life years (QALYs), the most important outcome from the health economics point of view [60]. The five level (5 L)-version is preferred over the three level (3 L)-version as it is more nuanced and thus more sensitive to gains (or losses) in general health [62]. The EQ-5D-5L will be assessed at baseline and both follow-up moments and will additionally be collected after the final treatment session.

- Productivity Cost Questionnaire (*i*PCQ) [63]

The iMTA Productivity Cost Questionnaire (*i*PCQ) includes three modules measuring productivity losses of paid work due to (1) absenteeism and (2) presenteeism (productivity loss due to paid work) and productivity losses related to (3) unpaid work. The *i*PCQ delivers the necessary input to calculate productivity loss. *i*PCQ will be collected monthly, meaning every 3 to 5 weeks. To organise this systematically, patients of the intervention group will be asked to complete this questionnaire at treatment session two, four and six and patients of the control group at treatment session four, eight and twelve. Both groups will additionally receive the *i*PCQ at 1 month follow-up and 3 months follow-up, as defined above. An additional two-month follow-up moment is planned for the *i*PCQ (range: 3 to 5 weeks after 1 month follow-up assessment).

##### Diary of medical costs

As patients are allowed to seek medical help outside the participating hospital between the 1 month and 3 months follow-up visits, healthcare/medication use cannot be monitored using the hospital’s financial records. Therefore, patients will be handed a diary in which they should collect all information regarding tinnitus related medical help, such as physician consultations (including general practitioners), psychological consultations, medication use and treatments in the context of their tinnitus complaints.

### Ethics

Every patient will impart written consent for participation. The study protocol and the use of the developed smartphone application received a consolidated positive advice by the Belgian Federal Agency for Medicine and Health Products (FAMHP) and an independent Ethical Committee (Ref. CIV-BE-21-09-037760). The study protocol was registered at Clinicaltrials.gov (ref. NCT05245318).

### Intervention

#### Standard clinical care (control treatment)

The standard care treatment comprises 12 face-to-face individual physiotherapy sessions at a ratio of 1 session of 30 min a week [14, 37]. Every patient in the control group will be treated in a tailored manner according to their results on the baseline measurements and will thus receive a personalised 12-week treatment program. The multimodal treatment program will consist of exercises to increase strength, endurance and coordination of the cervical spine (deep neck flexors and extensor muscles) and shoulder stabilising muscles, exercises to increase mobility and improve posture [14, 15]. In case of jaw complaints, stretching exercises of the masticatory muscles to increase jaw mobility are added to the program [15, 19]. In addition, manual mobilisations can be performed if limitations in the mobility of the neck or temporomandibular joints are observed. In the same manner, manual techniques to decrease muscle tension in neck and jaw muscles can be added to the exercise program [14, 15, 19]. Additionally, patients receive counselling to learn about their neck/jaw complaints and to guide them to good posture and movement habits, such as emphasising the importance to change posture when sedentary and specific advice concerning mouth habits and sleep hygiene in patients with jaw complains [15]. Patients will be motivated to perform the exercise program at home on a daily basis but will not receive additional support on the home exercises between sessions. The patient’s performance on the exercises will be evaluated during the weekly session in the clinic. Patients in the control group will not receive any tinnitus counselling.

#### Blended physiotherapy program (experimental treatment)

The experimental group will receive a blended physiotherapy program consisting of only six instead of twelve physiotherapy treatments over a period of 12 weeks, meaning one treatment session of 30 min every 2 weeks. In addition, a smartphone application will offer the patients a daily exercise program, based upon the standard physiotherapy treatment for ST [14–22], and psychological tinnitus counselling. This tinnitus counselling program consists of two sessions a week and is developed by psychologists of the University of Regensburg [64, 65]. Before the start of the program, the exercises will be tailored to the area, type and degree of the patient’s dysfunctions. During the six physiotherapy treatment sessions, patients will, if needed, receive manual mobilisations to increase mobility of the neck and/or temporomandibular joints or manual techniques to decrease muscle tension in neck and jaw muscles [14–22]. Additional counselling on knowledge about neck and jaw pain and advice about posture and movement habits will also be provided by the physiotherapist during these six treatment sessions.

#### Post-trial care

Patients of both intervention groups will be followed up by the multidisciplinary team of the University Hospital of Antwerp after the study trajectory and if requested by the patient post-trial care will be provided when necessary.

### Sample size and power

The study is designed as a non-inferiority trial of a continuous response variable comparing independent control and experimental subjects. The primary outcome is the change in TFI from baseline to 1-month follow-up. We hypothesise that there is no significant difference between the control and experimental group for this outcome. A non-inferiority margin is chosen at 6.5 points, being half of the minimally clinically important difference [66]. Assuming a standard deviation of 20 points (SD found in international literature [67] as well as in previous performed own studies [14, 18]) and a significance level (*α*) of 0.025, an achieved sample size of 150 patients per group is required to have 80% power to show non-inferiority using a one-sided, independent samples *t*-test. Considering a possible 10% drop-out, based on previous studies in ST [14, 37], 167 patients will be recruited into each group. Hence, a total of 334 patients will be recruited for the study.

### Data analysis and statistics

#### Effectiveness analysis

##### Primary analysis

Our primary hypothesis is that the change in TFI-score from baseline to 1-month follow-up will be non-inferior in the experimental group compared to the control group. After calculating a TFI-change score from baseline to 1-month follow-up, a two-sided 95% confidence interval for the mean difference in this change score between experimental and control group will be compared to the non-inferiority margin of 6.5.

##### Secondary analysis

The continuous secondary outcomes and baseline characteristics, measured at the different time points, will be used in a linear mixed model allowing correction for repeated measurements from the same patient to compare their profiles over time and identify at which time points the groups differ. Other variables such as age, gender and tinnitus grade can also be included in these models.

Considering this is a non-inferiority trial, primary and secondary endpoints are analysed in first instance in the per-protocol population. The per-protocol population are all eligible patients who adhered to the complete protocol. The intention-to-treat population will be taken into consideration in a sensitivity analysis afterwards.

#### Cost -effectiveness analysis (CEA)

The outcome of the CEA is the incremental cost-effectiveness ratio (ICER) of the blended physiotherapy program compared to the usual care program. The ICER is calculated as incremental costs divided by incremental effects. The analysis thus consists of two components: (1) incremental costs and (2) the incremental health outcomes (effects).

##### Costs

In accordance with the European Network for Health Technology Assessment (EUnetHTA) guidelines, the CEA will comprise two parts: a base case analysis, which only takes into account direct medical costs, and an extended analysis, which additionally considers relevant indirect costs such as work absenteeism and productivity loss [68]. As all relevant direct medical costs (i.e. consultation fees, scans, medication, etc.) stem from inpatient care provision, the costs for the base case analysis will be collected using the financial records of the Antwerp University Hospital. Relevant medication use is registered as a standard practice in the RCT described above. To assign costs to medication use, we will use the official unit prices of the Rijksinstituut voor Ziekte- en Invaliditeitsverzekering (RIZIV)/ l’Assurance soins de santé et indemnités (INAMI). As discussed above, patients are allowed to seek medical help outside the hospital between the two follow-up moments. As a consequence, healthcare and medication use cannot be monitored using hospital’s financial records in this period of time. Therefore, patients will keep a diary specifically designed for this study in which they should report all information regarding tinnitus related medical help and medication use. The content of this diary is discussed elaborately above.

Apart from the direct medical costs, the CEA will also incorporate relevant indirect costs in a separate, extended analysis. The abovementioned *i*PCQ will be used to collect the necessary input to calculate the costs associated to absenteeism and productivity loss due to tinnitus complaints following the Human Capital Approach (HCA) [68]. The HCA values the time during the whole period of work inability due to sick leave by multiplying the total number of days of work absenteeism by the national average labour cost per day. To calculate the productivity loss at work, the number of days with productivity loss will be multiplied by efficiency loss and the daily average of hours worked. Next, to value the productivity loss, this result will be multiplied by the national average labour cost per hour. All cost data will be collected over the entire study period starting at baseline.

##### Health outcomes

To evaluate the potential health gains of ST patients due to the blended physiotherapy program, this study will employ quality-adjusted life years (QALYs). QALYs combine both length of life (LoL) and health-related quality of life (HRQOL) in one composite measure. One QALY gained because of the blended physiotherapy program can thus be interpreted as living one additional year in perfect health due to the proposed treatment.

HRQOL will be determined using the EuroQoL EQ-5D-5L questionnaire, which is discussed elaborately above. While we expect HRQOL to be equal across groups at visit 1, data collection at baseline is necessary to be able to adjust for possible, but unlikely, baseline differences between the control group and intervention group. The resulting health states will be converted into health utilities (ranging from 0 to 1 with 0 equal to death and 1 to perfect health) using the recently published utility value sets for Belgium and will at its turn be used to calculate QALYs [61]. Next, incremental health gains will be calculated by comparing the QALYs of the intervention group to the control group.

#### Cost-effectiveness

Finally, the ICER will be calculated by dividing incremental costs by incremental health gains and is by consequence expressed as €/QALY gained. This outcome can thus be interpreted as the additional amount of costs needed for the blended physiotherapy program to gain one additional QALY compared to the usual care program. The robustness of the results will be assessed by bootstrapping (> 1000 samples) and be presented on a cost-effectiveness plane. The results will consist of a base case analysis taking into account only the direct medical costs and an extended analysis taking into account both direct and indirect costs.

## Discussion

The aim of this study is twofold, namely investigating the effectiveness and cost-effectiveness of a blended physiotherapy program for ST, including a smartphone application designed to increase therapy compliance and provide tinnitus counselling.

The study population in scope are adult ST patients. Patients will be diagnosed with ST by a multidisciplinary team of specialists in their field. This team will exist of ENT doctors, audiologists, physiotherapists and psychiatrists. The ENT doctors and audiologists will examine the presence of possible hearing loss, underlying disorders and tinnitus. The physiotherapists will assess the presence of neck and jaw dysfunctions and the link between these dysfunctions and the tinnitus using the diagnostic criteria for somatic tinnitus [31]. Any active psychiatric disorders, such as anxiety or depression disorders, will be excluded by the psychiatrist. This extensive diagnostic process by a multidisciplinary team is a major strength of our study.

The main scientific risks associated with the present study protocol are insufficient enrolment and patient drop-out. However, every year 2500 patients suffering from tinnitus seek help at the ENT department at UZA. As a quarter of these patients are diagnosed with ST, a maximum of 625 patients will be available for inclusion in our study on an annual basis. Therefore, we expect sufficient enrolment during the inclusion period of 2.5 years. In addition, the willingness of participants to complete the full treatment program and all follow-up tests is crucial for the power of the study results. The risk for drop-out is especially high in patients who do not experience the expected treatment results. To maximise treatment adherence and minimise drop-out, patients will be encouraged to complete the treatment and follow-up phase by the therapist, the study nurse and the researcher. Nonetheless, a drop-out of 10% was considered in the sample size calculation.

This study will use a high-quality methodological design to perform a single blind two-arm randomised controlled trial. A total of 334 patients will be randomised into two groups and the current standard care will be compared to the use of a blended physiotherapy program. In studies investigating physiotherapy treatment, blinding of subjects and therapists is typically difficult. In our study, patients will be blinded towards the group they will be randomised to, but the therapist cannot be blinded, as she knows whether a patient receives the treatment every week or every 2 weeks. To limit the risk of bias related to lack of blinding of the therapist, all evaluation measurements will be performed by a blinded assessor. All physiotherapy sessions will be provided in the clinic by the same therapist. This approach was chosen to ensure all patients will receive the same treatment and to limit therapist-dependent differences in outcome in our study. This approach might decrease the transferability of our study results to a clinical situation but was deemed necessary to avoid bias.

The proposed approach of the blended physiotherapy program using a smartphone application offering both an exercise program and tinnitus counselling has never been used before in ST treatment. Similar app-based programs, however, have proven to be effective and feasible for the treatment of neck pain [69, 70]. There is also an increasing interest in the use of smartphone applications for tinnitus patients [71, 72]. This study combines both tinnitus counselling and neck/jaw treatment providing a digital application to empower ST patients to improve and better manage their own health. Earlier research has already proven that the content of the application, both the exercise program and the counselling, is effective when used in a clinical face-to-face setting [14–22, 73, 74]. However, it was never examined in the context of a smartphone application as part of a blended physiotherapy treatment. The use of our developed smartphone application is expected to help overcome common hurdles related to the in-clinic treatment of patients with ST.

In standard care, patients with somatic tinnitus receive weekly face-to-face therapeutic sessions of 30 min to reduce their neck and jaw complaints in order to also alleviate the tinnitus. On average, 9 to 12 weeks are needed to achieve the desired effect [13, 34]. In addition, patients are allways recommended to perform exercises at home. This has proven to be effective in decreasing neck pain and avoiding recurrence of the complaints, but only if patients adhere [75, 76]. Compliance to home exercises is often impeded reducing positive long-term effects of the treatment [75–80]. This is not only the case for the treatment of patients with neck complaints, but a general hurdle in musculoskeletal treatment. Reasons for low compliance to home exercises are mostly investigated in studies on low back pain but are expected to be similar for other muscoloskeletal dysfunctions [77, 79]. The most important defined barrier was ‘lack of support’, as patients indicated they did not receive sufficient instructions and follow-up to perform the exercises correctly and consistently [75, 77]. Other contributing factors are patient attitudes and beliefs, self-efficacy, locus of control, stage of change and psychosocial issues [79]. The proposed blended physiotherapy program might give answer to some of the barriers experienced by standard care. The smartphone application will change patients locus of control and promote self-efficacy by providing sufficient support and clear instructions on how to perform the exercises correctly and regurlarly. The psychological counselling provided by the app will increase the knowledge of patients about tinnitus improving patient attitudes and believes about the treatment.

The current standard physiotherapy treatment is, although effective, costly and time consuming, especially in case of recurrent complaints where patients often need several follow-up consultations and additional treatment. The longevity of the need of treatment due to recurrent complaints leads to high direct health care costs [8, 9]. Furthermore, also indirect costs, such as work absenteeism and productivity losses are high in these patients [8, 9]. Our investigated blended physiotherapy program aims to decrease the patient’s need for in-clinic treatment, without reducing the effectiveness of the treatment. The alleviation of tinnitus improves the quality of life and, as a consequence, is expected to increase productivity and reduce absenteeism. In addition, the blended physiotherapy program will reduce the need for face-to face clinical care and is expected to improve the patient's self-management skills, rather than depending on the therapist’s care in case of recurrent symptoms. All these factors are expected to contribute to a significant decrease in both direct and indirect costs due to somatic tinnitus. The proposed blended physiotherapy program has the potential to be a valuable alternative for the current standard care, with a better cost-effectiveness without decreasing the effectiveness of the treatment.

## Trial status

This is the first version of the protocol registered on 26 January 2022 at Clinicaltrials.gov: NCT05245318. The trial is currently (February 2022) in the starting phase of recruitment and is anticipated to be completed in December 2025. In case of a substantial amendment to the protocol, this will be altered through clinicaltrials.gov.

## Supplementary Information

**Additional file 1.** A completed SPIRIT checklist 2013: Recommended Items to address in a clinical trial protocol and related documents (Ethical approval document and funding documentation (copy of original and English translation)) [30].

## Data Availability

As stated by the SPIRIT guidelines, the authors assure that breaking of the blind will occur at the end of the study and that data that break the blind will not be presented before release of mainline results. Data collected within the study will be disseminated to the public through scientific publications (according to the CONSORT guidelines), conferences and lectures. Results will be reported regardless of the outcome of the study. The raw data will be stored in RedCap, a secure web platform for building and managing online databases and surveys used at UZA. After publication of the results, an anonymous version of the raw data will be made available via an online repository server upon motivated request. The data management team consist of WS, SM, AG and VVR. The project will also be monitored by an independent study monitoring agency. In addition, there will be an e board of General practitioners, physiotherapists, audiologists, psychologists and patients.

## Abbreviations

CATT: Cost-effectiveness of a smartphone Application for Tinnitus Treatment
ST: Somatic or Somatosensory Tinnitus
RCT: Randomised controlled trial
ENT: Ear Nose Throat
UZA: University Hospital Antwerp
TFI: Tinnitus Functional Index
HRQOL: Health-related quality of life
TMJ: Temporomandibular joint
SPIRIT: Standard Protocol Items Recommendations for Interventional Trials
FUV: Follow-up visit
EQ-5D: EuroQoL-5 dimensions
VAS: Visual analogue scale
NVA: Nederlandse Vereniging Audiologie
SPIQ: Speech in quiet
SPIN: Speech in noise
LIST: Leuven Intelligibility Sentences Test
SRT: Speech reception threshold
NBQ: Neck Bournemouth Questionnaire
TMD: Temporomandibular disorder
HADS: Hospital Anxiety and Depression Scale
HQ: Hyperacusis Questionnaire
iPCQ: Productivity Cost Questionnaire
FAHMP: Federal Agency for Medicine and Health Products
CEA: Cost-effectiveness analysis
ICER: Incremental cost-effectiveness ratio
EUnetHTA: European Network for Health Technology Assessment
RIZIV: Rijksinstituut voor Ziekte- en Invaliditeitsverzekering\
INAMI: l’Assurance soins de santé et indemnités
QALYs: Quality-adjusted life years
LoL: Length of life
