# Supplementary material for: Cost-effectiveness of a smartphone Application for Tinnitus Treatment (the CATT trial): a study protocol of a randomised controlled trial
Source: Trials. 2022 May 23;23:435. doi: 10.1186/s13063-022-06378-7 (PMC9125968; doi:10.1186/s13063-022-06378-7)
Supplement: Supplementary file 1 — Additional file 1. A completed SPIRIT checklist 2013: Recommended Items to address in a clinical trial protocol and related documents (Ethical approval document and funding documentation (copy of original and English translation)) [30]. [file 13063_2022_6378_MOESM1_ESM.zip › CIV-21-09-037760_Approval letter with condition[47].pdf]

Federal Agency for Medicines and Health Products
Avenue Galilée - Galileelaan 5/03
1210 BRUSSELS
www.famhp.be

Annemie Zenner
Tel.: +32 (0)2 528 40 00
e-mail: annemie.zenner@fagg-afmps.be

Hasselt University
Mrs Michiels
Agoralaan Building A
3590 Diepenbeek
Belgium

Your letter from	Your reference	Our reference	Annex	Date
		CIV-21-09-037760	2	06/12/2021

Onderwerp Goedkeuring van een klinisch onderzoek op 06/12/2021
Titre de l'objet Approbation d'une investigation clinique le 06/12/2021
Subject Authorization of a clinical investigation dated 06/12/2021

Cost-effectiveness of a smartphone Application for Tinnitus Treatment: The CATT trial

Eudamed: CIV-21-09-037760

Chère Madame, Cher Monsieur,

Conformément au règlement (UE) 2017/745 sur les dispositifs médicaux, il a été décidé d'autoriser l'investigation clinique mentionnée ci-dessus. Cependant, un suivi doit être apporté aux points mentionnés en annexe. Cette autorisation est le résultat d'avis favorables émis par l'AFMPS et un comité d'éthique désigné par le Collège en accord avec la loi du 22 décembre 2020. La liste des documents sur laquelle la décision est basée et la liste des sites et investigateurs approuvés sont en annexe de cette approbation.

Veuillez noter que tous les évènements indésirables graves doivent être immédiatement communiqués à notre service. En outre, nous devons être informés des résultats de cette investigation clinique.

Salutations sincères,

Pour le Ministre des Affaires sociales et de la Santé publique

Geachte Mevrouw, Geachte Heer,

In overeenstemming met de verordening (EU) 2017/745 betreffende medische hulpmiddelen werd er besloten het bovenvermeld klinisch onderzoek goed te keuren. Niettemin moet er gevolg gegeven worden aan de voorwaarden vermeld in bijlage. Deze goedkeuring is het resultaat van de gunstige adviezen verleend door het FAGG en het Ethisch Comité aangewezen door het College volgens de wet van 22 december 2020. De lijst van documenten waarop deze beslissing is gebaseerd en de lijst van goedgekeurde sites en onderzoekers bevinden zich in bijlage van deze goedkeuring.

Gelieve te noteren dat alle ernstige ongewenste voorvallen onmiddellijk ter kennis moeten gebracht worden van onze dienst. Tevens dienen wij op de hoogte te worden gehouden van de resultaten van dit klinisch onderzoek.

Met de meeste hoogachting,

Voor de Minister van Sociale Zaken en Volksgezondheid

Dr. Greet Much

Unofficial translation

In accordance with the regulation (EU) 2017/745 on medical devices it was decided to authorize the above mentioned clinical investigation. However, the conditions as mentioned in annex are to be followed up. This decision is the result of the advice issued by the FAMHP and an Ethics Committee designated by the College in line with the law of 22 December 2020. The list of documents on which the opinion was based and a list with approved sites and principal investigators can be found in annex.

Please note that all serious adverse events must be immediately notified to our service. Furthermore, we must be informed about the results of this clinical investigation.

Annex I

Condition

The clinical investigation cannot start if the condition defined is not met.

To fulfil the conditions, the EC would like to receive a signed commitment of the sponsor that

i) the ICF and protocol will be adapted as requested

and

ii) the ICF and protocol will be submitted, together with the new insurance certificate and the manual for patients, as a substantial modification for approval by the EC.

ICF dd 17Nov2021:

1. The version number in the foot note was not updated (this is still version 1.0). Please update the version number of the document. Please also make sure the correct version number and version date are presented in the "list of submitted documents".

2. P1-2/6; "Omdat de vragenlijsten waarvan hier sprake via een smartphone applicatie dienen ingevuld te worden, moet u in het bezit zijn van een smartphone en de basisvaardigheden hebben om die te gebruiken, als u aan de studie wil deelnemen. Het gebruik van deze applicatie is gratis, maar bij het downloaden van de applicatie zal u de gebruikersvoorwaarden van de applicatie moeten aanvaarden. Deze gebruikersvoorwaarden zijn dezelfde die ook voor deelname aan de studie gelden en hebben geen invloed op de verplichting van de opdrachtgever van de studie om uw persoonlijke gegevens te beschermen. Niemand buiten het team van de arts-onderzoeker zal toegang hebben tot uw persoonlijke gegevens."

➔ In RFI §21 we requested to provide this information about the application in the ICF. However, based on the reply on RFI §20 we assume that participants in the control group won't use an application, but will only receive a link?

➔ It is important that participants do not know to which group they are assigned? In that case, we believe the patients will be biased due to the mentioning of the application in the ICF. Therefore, we suggest to adapt the text and make it more generic, to avoid such bias:

"Omdat de vragenlijsten waarvan hier sprake is via een smartphone ~~applicatie~~ dienen ingevuld te worden, moet u in het bezit zijn van een smartphone en de basisvaardigheden hebben om die te gebruiken, als u aan de studie wil deelnemen. **De opdrachtgever is verantwoordelijk voor het gebruik van elk digitaal hulpmiddel (vb. website,**

applicatie,…) in een klinische studie. Dit wil zeggen dat de nodige analyses werden uitgevoerd om na te gaan of ze conform zijn met de GDPR wetgeving, namelijk dat het gebruik ervan gerechtvaardigd is, en bovenal veilig is. Bovendien dient elk digitaal hulpmiddel gratis te zijn voor deelnemers in een studie. Aan het gebruik van sommige digitale hulpmiddelen zijn algemene voorwaarden gekoppeld. In dat geval zal men u vragen om hier akkoord mee te gaan. ~~Het gebruik van deze applicatie is gratis, maar bij het downloaden van de applicatie zal u de gebruikersvoorwaarden van de applicatie moeten aanvaarden.~~ Deze gebruikersvoorwaarden doen geen afbreuk aan de verplichtingen van de opdrachtgever ten opzichte van de patiënt, in het bijzonder wat betreft de bescherming van de privacy van de patiënt. ~~zijn dezelfde die ook voor deelname aan de studie gelden en hebben geen invloed op de verplichting van de opdrachtgever van de studie om uw persoonlijke gegevens te beschermen.~~ Niemand buiten het team van de arts-onderzoeker zal toegang hebben tot uw persoonlijke gegevens."

3. RFI §22; According to the response to RFI §22 a manual was prepared for the patient with a clear description on how to use the application. Except mistaken, we did not receive this manual?

4. RFI §32: "32. *Please add in the ICF some information or advice to the respondents, found not to be suitable for inclusion in the clinical trial.*"

   Except mistaken, we could not find this information in the ICF. Please add some information for patients that cannot participate.

Protocol:

5. RFI §28; in the reply to RFI §28 it is indicated that it will not be able to identify patients based on only date of birth and gender.

   Taking into account the GDPR-principle of data minimization, we assume that it is sufficient to collect the birth year instead of the full date of birth?

6. Cfr. Comment §2 on the ICF; please update the protocol based on this information. Except mistaken no information was provided in the protocol about the RedCap link and the questionnaire that will be completed by the control group.

7. RFI §30; P18/20; "*The smartphone application will be made available by the manufacturer during the study, to study participants only, using a unique identifier code. The application will be made available for both Android and iOS devices. During the study, the manufacturer will maintain the application and updates will be made available if necessary, to make sure the application stays up and running after operating system updates. No other changes than those necessary for the good functioning of the application will be made after the start of the study.*"

   „EC would like to point out that the sponsor is responsible for the use of the Application. More specifically, the sponsor should conduct all essential GDPR checks to make sure the use of this application is justified, GDPR compliant and safe for the processing of sensitive personal data. The patient should be well informed about the terms and conditions of application. Furthermore, the sponsors obligations should not be compromised by these terms and conditions, especially in terms

of privacy protection of the patient. Please inform the patient about additionally costs (if any) that are linked to the usage of the application. This information should be added to the ICF, if necessary.

Finally, EC research emphasizes that only essential data for the purpose of the applications can be collected (cfr. GDPR principle of data proportionality and minimization).

Please add a statement to the protocol to confirm this. (Q)"

Except mistaken, the requested information was not added to the protocol.

Insurance:

8. RFI §35;"*The certificate should refer to the Law on medical devices d.d. 22 December 2020 (article 32) instead of the Law of 7 May 2004 concerning experiments on the human person. (Q)*"

   An adjusted insurance certificate was requested, but has not yet been received from the insurance company. ➔ Thank you for contacting the insurance company for a new certificate. Please submit the updated certificate once this is finalized.

Annex II

List of documents on which the advices are based

Documents	*Version and/or date*
**COVER LETTER**	
CoverLetter_20211117.pdf	V1.3 / 17-11-21
**APPLICATION FORM**	
ApplicationFrom_v1.2_20211109.pdf	V1.2 / 09-11-21
ApplicationFromSigned_v1.2_20211109.pdf	V1.2 / 09-11-21
**CIP**	
ClinicalInvestigationPlan_CIP_v1.1_20211109.pdf	V1.1 / 17-11-21<br>Not approved, see condition
**INVESTIGATOR'S BROCHURE (including any annexes)**	
InvestigatorBrochure_v1.2_20211117.pdf	V1.2 / 17-11-21
**MANUFACTURER'S INSTRUCTIONS FOR USE**	
Added to CIP	*See CIP*
**LIST OF GENERAL SAFETY AND PERFORMANCE REQUIREMENTS**	
ListGeneralSafety.pdf	V1.0 / 13-08-21
**NOTIFIED BODY CERTIFICATES**	
NA	
**PROOF OF INSURANCE**	
ProofInsurance.pdf	V1.0 / 27-08-21
**SUITABILITY OF SITES**	
Suitability of Site - UZA.pdf	V1.0 / 20-09-21
Suitability of Site - HasseltUniversity.pdf	V1.0 / 20-09-21
**EXAMPLE OF LABELS**	
Example of Lables_v1.1_20211117.pdf	V1.1 / 17-11-21
**DECISIONS FROM OTHER COUNTRIES**	
NA	
**PATIENT RELATED DOCUMENTS**	
INFORMED CONSENT_20211117.pdf	V1.1 / 17-11-21
ProceduresInclusionPatients_v1.1_20211117.pdf	V1.1 / 17-11-21
**CV OF PRINCIPLE INVESTIGATOR(S)**	
Investigator CV_AG.pdf	V1.0 / 16-09-21
Investigator CV_SMichiels.pdf	V1.0 / 16-09-21
Michiels_ManualTherapy_2016.pdf	V1.0 / 16-09-21
Declaration_interest_template_en_SMichiels.pdf	V1.0 / 16-09-21
**PMCF PLAN**	
NA	
**CEP**	
ClinicalEvaluationPlan_CEP_v1.1_20211117.pdf	V1.1 / 17-11-21
**CIP synopsis**	
CIPsynopsis_v1.1_20211117.pdf	V1.1 / 17-11-21
**Compliance with the applicable rules on the protection and confidentiality of personal data/ personal information**	
Compliance with the rules on Data Protection_v1.1_20211117.pdf	V1.1 / 17-11-21
**OTHER DOCUMENTS**	
Financial disclosure_AG.pdf	V1.1 / 17-11-21
ExpertPanelOpinion.pdf	V1.0 / 01-07-21

The Ethics Committee has reviewed all the participant's documents (e.g. ICF's, patient cards,…) in the following language(s):

- Dutch

The correctness of the translation of these participant's documents in other languages than mentioned above, is the responsibility of the sponsor.

Annex III

List of approved sites and investigators for the clinical investigation:

UZA – prof dr Annick Gilles

UHasselt – dr Sarah Michiels
