# Supplementary material for: Cost-effectiveness of a smartphone Application for Tinnitus Treatment (the CATT trial): a study protocol of a randomised controlled trial
Source: Trials. 2022 May 23;23:435. doi: 10.1186/s13063-022-06378-7 (PMC9125968; doi:10.1186/s13063-022-06378-7)
Supplement: Supplementary file 1 — Additional file 1. A completed SPIRIT checklist 2013: Recommended Items to address in a clinical trial protocol and related documents (Ethical approval document and funding documentation (copy of original and English translation)) [30]. [file 13063_2022_6378_MOESM1_ESM.zip › Funding documentation TBM-project FWO original.pdf]

Geachte professor Michiels,

De raad van bestuur van het FWO heeft uw aanvraag voor T002221N: Cost-effectiveness of a smartphone Application for Tinnitus Treatment: The CATT trial goedgekeurd. Graag wil ik u dan ook feliciteren met de toekenning van het door u aangevraagde onderzoeksproject voor een totale subsidie van 849.608,00 euro.

De toegekende toelage is beschikbaar per 1 oktober 2021 en dit voor de hele projectperiode, voor zover de financierende overheden hiervoor de nodige middelen blijvend vrijmaken en mits blijvend voldaan aan de reglementering van het FWO.

De dienst onderzoekscoördinatie/contractbeheer van uw onderzoeksinstelling zal kortelings van het FWO de steunovereenkomst(en) ontvangen via het FWO Extranet en instaan voor de ondertekening ervan door alle contractuele partijen. Deze steunovereenkomst wordt opgesteld volgens het reglement van de TBM-projecten van het FWO: Fonds Wetenschappelijk Onderzoek - Vlaanderen - Reglement Toegepast Biomedisch onderzoek met een primair Maatschappelijke finaliteit (TBM) (fwo.be)

Voor elk project dat een samenwerking omvat tussen **meerdere begunstigden**, dient u het FWO (tbm@fwo.be) binnen een periode van 4 maanden na ontvangst van de steunovereenkomst een door alle partijen ondertekende **samenwerkingsovereenkomst** te bezorgen. ***De ontvangst van deze samenwerkingsovereenkomst door het FWO is noodzakelijk voor de verdere uitbetaling van de subsidie.***

Het FWO verwacht tevens, uiterlijk 6 maanden na de officiële start van het project , een ingevuld Data Management Plan. Dit DMP moet op dat moment niet bij het FWO ingediend worden, maar wel bij de bevoegde dienst onderzoekscoördinatie van de onthaalinstelling. Meer informatie vindt u op onze website http://www.fwo.be/nl/het-fwo/organisatie/data-management-plan/.

Wat betreft de toepassing van de Algemene Verordening Gegevensbescherming m.b.t. de verwerking van de persoonsgegevens verwijs ik naar de desbetreffende bepalingen in de overeenkomst.

Jaarlijks dient u een vergadering te organiseren met het adviserend comité. ***Het FWO dient te worden uitgenodigd op deze bijeenkomst via tbm@fwo.be en bij afloop dienen de notulen van de vergadering aan het FWO te worden bezorgd.*** Gelieve steeds ook het projectnummer te vermelden bij deze communicatie.

Informatie omtrent de **rapporteringsmodaliteiten** vindt u op de website: https://www.fwo.be/media/839503/Guidance-TBM-follow-up-2018.docx

Tenslotte vraag ik u met aandrang om op uw publicaties steeds te vermelden dat ze tot stand kwamen met de financiële steun van het FWO. Vacatures gekoppeld aan onderzoeksprojecten kunnen bekend worden gemaakt via de FWO-website. U kan het vacaturebericht aan het FWO bezorgen via communicatie@fwo.be. Aanvullend kan het nuttig zijn om dergelijke berichten ook te publiceren via de Euraxess website van de Europese Commissie: http://ec.europa.eu/euraxess/.

Voor bijkomende inlichtingen kan u via tbm@fwo.be steeds terecht bij de dossierbeheerder voor vragen rond het TBM subsidieprogramma. Gelieve bij elke communicatie het bovenvermelde projectnummer te vermelden.
Vergeet ook niet het FWO te volgen via Twitter (@FWOVlaanderen) en LinkedIn om op de hoogte te blijven van de laatste ontwikkelingen en nieuwe oproepen!

Ik wens u zeer veel succes toe met de uitvoering van uw project.

Met vriendelijke groeten,

in opdracht van
dr. Hans Willems
Secretaris-generaal

Nena Van den Steen
Dossierbeheerder
TBM, Gedrags- en Maatschappijwetenschappen

FWO
Egmontstraat 5, 1000 Brussel

T +32 2 550 15 62
tbm@fwo.be
gm@fwo.be

www.fwo.be
fwo Fonds Wetenschappelijk Onderzoek
Vlaanderen
Opening new horizons
