# Supplementary material for: Cost-effectiveness of a smartphone Application for Tinnitus Treatment (the CATT trial): a study protocol of a randomised controlled trial
Source: Trials. 2022 May 23;23:435. doi: 10.1186/s13063-022-06378-7 (PMC9125968; doi:10.1186/s13063-022-06378-7)
Supplement: Supplementary file 1 — Additional file 1. A completed SPIRIT checklist 2013: Recommended Items to address in a clinical trial protocol and related documents (Ethical approval document and funding documentation (copy of original and English translation)) [30]. [file Funding document translation.pdf › Funding document translation.pdf]

Dear professor Michiels,

The board of directors of the FWO has approved your application for T002221N: Cost-effectiveness of a smartphone Application for Tinnitus Treatment: The CATT trial. I would like to congratulate you with the award of your research project for a total subsidy of 849608 euro.

The grant will be available from October 1, 2021 for the entire project period, provided the funding authorities continue to make the necessary resources available and provided the regulations of the FWO continue to be complied with.

The research coordination/contract management department of your research institution will shortly receive the support agreement(s) from the FWO via the FWO Extranet and ensure that they are signed by all contracting parties. This support agreement is drawn up according to the FWO's TBM project regulations: Fonds Wetenschappelijk Onderzoek- Vlaanderen- Reglement Toegepast biomedisch onderzoek met een primair Maatschappelijke finaliteit (TBM) (fwo.be)

For any project involving a summary between multiple beneficiaries, please provide the FWO (tbm@fwo.be) with a cooperation agreement signed by all parties within a period of 4 months from receipt of the support agreement. The receipt of this cooperation agreement by the FWO is necessary for the further payment of the grant.

The FWO also expects a completed Data Management Plan (DMP) no later than 6 months after the official start of the project. This DMP does not have to be submitted to the FWO at that moment, but to the competent research coordination office of the receiving institution. More information can be found on our website http://www.fwo.be/nl/net-fwo/organisatie/data-management-plan/.

Regarding the application of the General Data Protection Regulation with respect to the processing of personal data, I refer to the relevant provisions in the agreement.

Annually, you should organize a meeting with the advisory committee. The FWO should be invited to this meeting via tbm@fwo.be and the minutes of the meeting should be provided to the FWO at the end. Please always mention the project number with this communication.

Information about the reporting modalities can be found on the website https://www.fwo.be/media/839503/Guidance-TBM-follow-up-2018.docx.

Finally, I urge you to always mention on your publications that they were realized with the financial support of the FWO. Vacancies linked to research projects can be announced via the FWO website. You can send the vacancy notice to the FWO via communicatie@fwo.be. It may also be useful to publish such announcements on the Euraxess website of the European Commission: https://ec.europa/euraxess/.

For additional information you can always contact the file manager for questions about the TBM subsidy program via tbm@fwo.be. Please mention the project number above in every communication. Do not forget to follow the FWO on Twitter (@FWOVlaanderen) and LinkedIn to stay informed about the latest developments and new calls!

I wish you a lot of success with the execution of the project.

Kind regards,

By order of Dr. Hans Willems

Secretary General

Nena Van den Steen
Dossierbeheerder
TBM, Gedrags- en Maatschappijwetenschappen

FWO
Egmontstraat 5, 1000 Brussel

T +32 2 550 15 62
tbm@fwo.be
gm@fwo.be

www.fwo.be

fwo Fonds Wetenschappelijk Onderzoek Vlaanderen
Opening new horizons

Translated with www.DeepL.com/Translator (free version)
